# Uterine Weight as a Modifier of Black/White Racial Disparities in Minimally Invasive Hysterectomy Among Veterans with Fibroids in the Veterans Health Administration

**DOI:** 10.1089/heq.2022.0130

**Published:** 2022-12-16

**Authors:** Cathea M. Carey, Jodie G. Katon, Andrew S. Bossick, Kristen E. Gray, Kemi M. Doll, Alicia Y. Christy, Lisa S. Callegari

**Affiliations:** ^1^ Health Services Research and Development (HSR&D) Center of Innovation for Veteran-Centered and Value-Driven Care, U.S. Department of Veterans Affairs, VA Puget Sound Healthcare System, Seattle, Washington, USA.; ^2^ Department of Health Systems and Population Health, University of Washington, Seattle, Washington, USA.; ^3^ Public Health Sciences, Henry Ford Health, Detroit, Michigan, USA.; ^4^ Department of Obstetrics and Gynecology, University of Washington, Seattle, Washington, USA.; ^5^ Women's Health Services, Veterans Administration, U.S. Department of Veterans Affairs, Washington, District of Columbia, USA.

**Keywords:** minimally invasive hysterectomy, racial disparities, uterine fibroids, Veterans, fibroid size

## Abstract

**Introduction:**

Uterine fibroids are the most common indication for hysterectomy. Minimally invasive hysterectomy (MIH) confers lower risk of complications and shorter recovery than open surgical procedures; however, it is more challenging to perform with larger fibroids. There are racialized differences in fibroid size and MIH rates. We examined the role of uterine size in black-white differences in MIH among Veterans in the Department of Veterans Affairs (VA).

**Methods:**

Using VA clinical and administrative data, we conducted a cross-sectional study among black and white Veterans with fibroids who underwent hysterectomy between 2012 and 2014. We abstracted postoperative uterine weight from pathology reports as a proxy for uterine size. We used a generalized linear model to estimate the association between race and MIH and tested an interaction between race and postoperative uterine weight (≤250 g vs. >250 g). We estimated adjusted marginal effects for racial differences in MIH by postoperative uterine weight.

**Results:**

The sample included 732 Veterans (60% black, 40% white). Postoperative uterine weight modified the association of race and MIH (*p* for interaction=0.05). Black Veterans with postoperative uterine weight ≤250 g had a nearly 12-percentage point decrease in MIH compared to white Veterans (95% CI −23.1 to −0.5), with no difference by race among those with postoperative uterine weight >250 g.

**Discussion:**

The racial disparity among Veterans with small fibroids who should be candidates for MIH underscores the role of other determinants beyond uterine size. To eliminate disparities in MIH, research focused on experiences of black Veterans, including pathways to treatment and provider-patient interactions, is needed.

## Introduction

Uterine fibroids (UF) are a common gynecological condition with well-documented racial disparities in incidence, severity, treatment, and outcomes.^1^ By age 50, ∼80% of black people and 70% of white people with a uterus will have UF, with up to 30% resulting in symptoms that require treatment.^2^ Not only do black people with a uterus have a higher prevalence of UF than their white counterparts, they also experience earlier onset, larger and more numerous UF, more severe symptoms, and have higher rates of surgical treatment, particularly hysterectomy.^1,3^ Although minimally invasive approaches for hysterectomy (vaginal or laparoscopic with or without robot assist) are preferred when possible due to shorter recovery times and decreased risk of complications, black people with UF who undergo hysterectomy are more likely than their white counterparts to have an open abdominal hysterectomy versus a minimally invasive hysterectomy (MIH).^4–6^

**FIG. 1.**
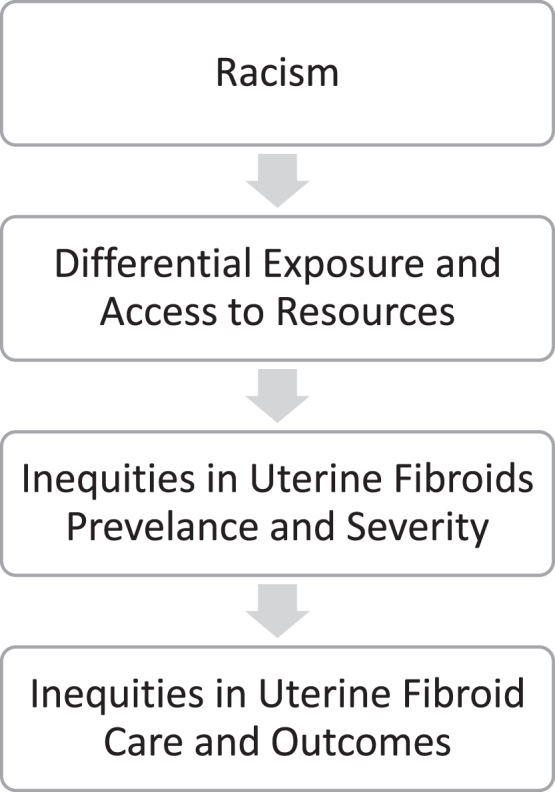
Pathway of the health impacts of multilevel racism on uterine fibroids treatment.

The disproportionate impact of UF on black people with a uterus, compared to their white counterparts, is a product of multilevel racism (structural, institutional, interpersonal)^7,8^ leading to differential lifetime and intergenerational exposures thought to influence UF (Fig. 1).^9^ Compared to white people, black people are disproportionately exposed to a range of factors thought to contribute to the etiology of UF and poorer UF outcomes,^10–12^ including poor diet,^13^ psychosocial stress stemming from racism,^14^ physical and sexual abuse,^15^ lower access to quality gynecologic care,^16^ and biased treatment within health care settings.^9^ These racialized patterns of exposures may also explain why black people compared to white people tend to have more advanced fibroid disease as evidenced by larger and more numerous UF at the time of hysterectomy.^3^

More advanced fibroid disease at the time of hysterectomy may also result from delayed treatment due to black patients being less likely to be presented with treatment options that align with their cultural preferences.^17,18^ Larger and more numerous fibroids are frequently posited as an explanation for lower rates of MIH among black patients,^19^ as MIH is more technically challenging with larger uterine size.^20^ However, other factors may also have a role in whether or not MIH is performed, including availability of trained surgeons, access to appropriate equipment and operative assists, and a patient's past surgical history, including presence of adhesive disease or scarring.^19,21–23^ Examining whether racial disparities in MIH are similar among those with smaller versus larger uterine size as approximated by postoperative uterine weight can shed light on factors underlying racial disparities in MIH eligibility beyond fibroid size and number.

Our prior work demonstrates the presence of racial disparities in MIH within the U.S. Department of Veterans Affairs (VA) Healthcare System,^22,23^ despite decreased barriers to access as many Veterans qualify for free care or have a nominal copay.^24^ We sought to build on these findings by determining whether uterine size (estimated by postoperative uterine weight) modified the association between race and MIH among black and white Veterans with UF undergoing hysterectomy in VA. We hypothesized that, if uterine size was the sole driver of the racial disparity in MIH, we would only observe racial differences in MIH among those with larger but not smaller uterine size.

## Methods

The study population was a subset of a larger cohort of Veterans who received a hysterectomy provided by or paid for by VA between fiscal year (FY) 2008 and FY 2014 (*N*=7906) identified using *International Classification of Diseases, Ninth Revision* (ICD-9) procedure codes, as described previously.^25,26^ This cross-sectional analysis included the subset of Veterans who had a hysterectomy provided by VA and a diagnosis of UF between October 1, 2012 and September 30, 2014.^27^ We excluded hysterectomies performed by providers in the community but paid for by VA as pre- and postoperative clinical documentation necessary for analysis was not consistently available for these surgeries.

We included only those whose reported race was listed as black or white in the VA administrative data to be able to place our findings in the context of existing literature of black/white disparities in rates of MIH and because black people with uterus are overrepresented in military service and the Veteran population compared to civilian population.^28,29^

The cohort included VA clinical and administrative data available from the VA Corporate Data Warehouse (CDW) as well as chart abstracted data from VA electronic medical record. Chart abstraction captured key clinical information used in surgical decision-making that was not available in structured fields in CDW (e.g., postoperative uterine weight). A trained abstractor reviewed electronic medical charts using a standard abstraction form and protocol and entered relevant data into a REDCap database.^30^ A board-certified VA gynecologist (L.S.C.) assisted with the development of the form, addressed questions that arose during the data collection process, and adjudicated items for the abstractor.^23,27^ This study was approved by VA Puget Sound Institutional Review Board.

### Outcome

The primary outcome was receipt of MIH, including all vaginal and laparoscopic/robot-assisted hysterectomies (ICD-9 68.59, 68.79, 17.41–17.44, and 17.49), versus abdominal hysterectomy (ICD-9 68.39, 68.49, and 68.69).^22^ The ICD-9 codes for distinguishing MIH versus abdominal hysterectomies were validated using chart abstracted surgical notes as the gold standard. ICD-9 codes had a sensitivity of 98%, specificity of 89%, and a positive predictive value of 85% for distinguishing MIH from abdominal hysterectomy (data not shown).

### Exposure and effect modifier

The exposure of interest was defined as black or white race based on CDW data, which relies on a combination of patient self-report and clerk entry and has been validated in prior VA research.^28^ Our effect modifier was uterine size as estimated by postoperative uterine weight dichotomized as ≤250 or >250 g abstracted from pathology reports. The cutoff point was based on surgical rationale for eligibility of MIH.^31^ We used postoperative uterine weight rather than preoperative ultrasound as this was more consistently recorded in the VA electronic health record. In addition, prior research demonstrates that postoperative uterine weight is a reliable proxy for preoperative uterine volume estimated from ultrasound (*r*=0.82).^32^

### Sample characteristics

To describe our sample's characteristics, we analyzed additional variables, including sociodemographics, general health, gynecologic, and reproductive history variables, which were ascertained from both administrative data and chart abstraction. Sociodemographics variables were obtained from administrative data, including age at the time of hysterectomy (18–39, 40–44, 45–49, ≥50 years), geographic region (West, Midwest, South, Northeast), and the calendar year, in which the surgery occurred (2012, 2013, 2014).^32^ General health indicators were also obtained from administrative data, including body mass index (BMI; <24.9, 25–29.9, ≥30 kg/m^2^),^34^ medical comorbidities defined by the Charlson comorbidity index using ICD-9 codes in the year before hysterectomy (none: 0, moderate: 1, or severe: ≥2),^27,35^ and mental health comorbidities defined dichotomously based on the presence of ≥1 diagnostic code in the year before hysterectomy for post-traumatic stress disorder, depression, and drug abuse or dependence as described in previous studies.^22,27^

Gynecologic conditions including pelvic pain/endometriosis and pelvic floor disorder/pelvic prolapse were identified by ICD-9 codes reported within the year before hysterectomy.^23,26^ Finally, we included additional gynecologic and reproductive history variables abstracted from the medical record, including parity and history of cesarean section (nulliparous; parous, no history of cesarean; parous, history of cesarean); prior myomectomy; and other prior abdominal surgery, excluding cesarean section.

### Statistical analysis

We described Veteran characteristics by race (black or white) and postoperative uterine weight (≤250 g vs. >250 g). We then calculated unadjusted percentages of MIH by race and postoperative uterine weight. To test whether postoperative uterine weight modified the association between race and MIH, we estimated a generalized linear model with a logit link and binomial distribution, including an interaction term between race and postoperative uterine weight. We accounted for nonindependence in site of care by clustering on VA medical facility.^36^ Our model only adjusted for FY of hysterectomy as all other measured variables were considered to be on the causal pathway between race (i.e., racism) and surgical mode (Fig. 1).^37–39^ We tested the statistical significance of the interaction term via Wald test.^36^

To facilitate interpretation, we used Stata's “margins” command using recycled predictions to obtain predictive margins (estimated probabilities of MIH) across groups defined by race and postoperative uterine weight and to obtain marginal effects of race (difference in probabilities of MIH between black and white Veterans) within each group defined by uterine weight.^36^ These adjusted probabilities were then used to estimate adjusted percentages and percentage point differences between group. To account for lack of a standard cut-point for postoperative uterine weight and examine the impact of varying this cut-point on our findings, we repeated all analyses ±50 g as alternative cut-points. All analyses were conducted using Stata MP 17^40^ and all statistical tests used an alpha of 0.05.

## Results

We sequentially excluded patients with unknown/missing race or a race other than black or white (*n*=94), Latinx Veterans (*n*=62), those with a gynecologic malignancy (*n*=73), those with an unspecified route of hysterectomy (*n*=0), and those whose postoperative uterine weight was deemed to be biologically implausible based on prior studies (*n*=105).^27^ The final analytic cohort consisted of *N*=732 Veterans (black *n*=439; white *n*=293). Fifty-four percent of black Veterans and 25% of white Veterans had a postoperative uterine weight >250 g. Among all Veterans, the percentage with MIH was lower among Veterans with postoperative uterine weight >250 g compared to those with postoperative uterine weight ≤250 g (23% vs. 60%).

Table 1 presents sample characteristics by race and postoperative uterine weight. Regardless of postoperative uterine weight, black and white Veterans with UF were similar in terms of most measured sociodemographic, general health, and gynecological, reproductive, and surgical history characteristics. However, among those with large and small postoperative uterine weight, black Veterans were more likely than white Veterans to live in the Southern United States (≤250 g: 69% vs. 46%; >250 g: 72% vs. 54%), more likely to have had a prior myomectomy (≤250 g: 11% vs. 4%; >250 g: 13% vs. 5%), and less likely to have a BMI <25 kg/m^2^ (≤250 g: 16% vs. 26%; >250 g: 11% vs. 30%).

**Table 1.** Characteristics of Black and White Veterans with Uterine Fibroids with a Veterans Affairs Provided Hysterectomy Between Fiscal Year 2012–2014, by Postoperative Uterine Weight (*N*=732)

	Postoperative uterine weight ≤250 g, *N*=423				Postoperative uterine weight >250 g, *N*=309			
	Black Veterans (*N*=204)		White Veterans (*N*=219)		Black Veterans (*N*=235)		White Veterans (*N*=74)	
	*N*	%	*N*	%	*N*	%	*N*	%
Sociodemographics								
Age								
18–39	50	25	52	24	38	16	10	14
40–44	65	32	49	22	59	25	19	26
45–49	57	28	56	26	88	37	27	36
≥50	22	11	34	16	50	21	18	24
Region								
West	31	15	56	26	25	11	14	19
Midwest	25	12	39	18	30	13	12	16
South	140	69	101	46	167	72	40	54
Northeast	8	4	23	11	11	5	8	11
Calendar year								
2012	27	13	27	12	21	9	2	3
2013	95	47	108	49	134	57	46	62
2014	82	40	84	38	80	34	26	35
General health indicators								
Charlson comorbidity index								
0	99	49	95	43	112	48	37	50
1	61	30	77	35	64	27	18	24
≥2	44	22	47	21	59	25	19	26
≥1 Mental health comorbidities^a^	124	61	130	59	167	71	48	65
Body mass index, kg/m^2^								
<25	32	16	57	26	26	11	22	30
25–29	61	30	51	24	74	32	19	26
≥30	110	54	109	50	134	57	33	45
Gynecological, reproductive, and surgical history								
Endometriosis/pelvic pain	155	76	148	68	150	64	50	68
Prolapse/pelvic floor disorder	22	11	44	20	15	6	9	12
Parity and history of cesarean section								
Nulliparous	34	17	48	22	60	26	31	42
Parous with history of cesarean	44	22	54	25	65	28	13	18
Parous with no history of cesarean	126	62	117	53	110	47	30	41
Prior myomectomy	23	11	8	4	30	13	4	5
Prior abdominal surgery	21	10	20	9	13	6	7	9

^a^ Composite of any diagnosis of post-traumatic stress disorder, depression, and drug abuse or dependence.

Among Veterans with postoperative uterine weight ≤250 g, the unadjusted percentage of black Veterans with MIH was lower than white Veterans (54% vs. 66%; Table 2). However, among those with postoperative uterine weight >250 g, the unadjusted percentage with MIH was higher among black Veterans compared to white Veterans (25% vs. 18%). In the generalized linear model for MIH, the interaction term between race and postoperative uterine weight was marginally statistically significant (*p*=0.05).

**Table 2.** Unadjusted and Adjusted Percentage of Veterans with Minimally Invasive Hysterectomy and Racial Differences in Adjusted Percent with Minimally Invasive Hysterectomy by Postoperative Uterine Weight

Postoperative uterine weight, g	Race	Unadjusted % with MIH	Adjusted % with MIH (95% CI)	Racial differences in adjusted % with MIH (95% CI)
	Black	53.9	53.9 (40.8 to 67.0)	−11.8 (−23.1 to −0.5)^a^
	White	65.8	65.7 (56.1 to 75.3)	
>250	Black	24.7	24.6 (15.9 to 33.4)	6.9 (−5.0 to 18.8)
	White	17.6	17.7 (6.0 to 29.5)	

^a^ *p*<0.05.

CI, confidence interval; MIH, minimally invasive hysterectomy.

After adjustment for FY of hysterectomy, among Veterans with postoperative uterine weight ≤250 g, black Veterans had a lower percentage of MIH compared to white Veterans (difference=−12 percentage points; 95% CI −23 to 0.5). Among Veterans with postoperative uterine weight >250 g, there was not a detectable difference in MIH between black and white Veterans (difference=6.9 percentage points; 95% CI −5.0 to 18.8). Results did not change when we used alternative cut-points for postoperative uterine weight (results not included).

## Discussion

In this study of over 700 Veteran VA users with UF, we found that black Veterans were more likely than white Veterans to have larger uterine size at the time of hysterectomy as estimated by postoperative uterine weight and that the association of race with MIH varied by uterine size. Specifically, while no statistically significant difference in MIH was detected between black versus white Veterans with larger uterine size, we observed a lower predicted probability of MIH in black versus white Veterans among those with smaller uterine size.

Our finding that black Veterans with UF were more likely than white Veterans with UF to have larger uterine size is consistent with prior literature.^41^ Previously, Kjerulff et al reported that, compared with white women undergoing hysterectomy for UF, black women had ∼100 g larger mean postoperative uterine weight (420.8 g vs. 319.1 g).^42^ Prospective data also indicate that black people experience earlier onset of fibroids and larger and more numerous fibroids than their white counterparts.^2,43^ Taken together, these results underscore the greater burden and potential health impacts of UF among black people with uteruses. However, these differences in UF cannot be explained by genetic variations in socially constructed racial categories.^41,44^

Rather, the large body of evidence linking the social, political, and environmental disadvantages perpetuated by structural racism to adverse health outcomes suggests that observed differences in fibroid pathogenesis are a result of downstream effects of racism, such as alterations in gene expression and sympathetic nervous system activation.^45^ In addition, larger and/or more numerous fibroids at the time of hysterectomy among black people may result from other factors, such as delays in care related to the normalization of symptoms by patients^46^; dismissal of symptoms and/or biased care by providers, including failure to provide culturally preferred treatment options^17,18,47,48^; or hysterectomy at a later stage of the disease due to differential preferences for uterine or fertility-preserving treatments.^49^

Our findings regarding variation in racial disparities in MIH by postoperative uterine weight are novel. Prior studies of racial disparities in MIH that have considered postoperative uterine weight have adjusted for it as a potential confounding factor.^31^ However, acknowledging the racial disparity in fibroid size and/or number at the time of hysterectomy as a downstream consequence of racism and an indicator of the context in which fibroids develop and are treated, we considered postoperative uterine weight as a potential effect modifier rather than as a confounder.^50^ Among those with larger postoperative weight, there were no significant differences in MIH by race.

In contrast, among Veterans with smaller postoperative weight who were likely appropriate candidates for MIH, we observed that black Veterans were less likely to have an MIH. This aligns with other research identifying racial disparities among candidates likely eligible for MIH and supports our hypothesis that additional factors beyond fibroid size and number contribute to racial disparities in MIH.^21^ Differences in UF treatment pathways between black/white Veterans could also influence racial disparities in MIH observed in our study. Specifically, prior procedural/surgical treatments for UF were more common among black Veterans and may have made them poorer candidates for MIH. In addition, regional differences in resources or practice patterns at VA facilities where black Veterans receive treatment or differences in interactions with clinicians, including quality of provider-patient communication and clinician bias, may also contribute to our findings.

Strengths of this study include the ability to explore associations between race, postoperative uterine weight, and MIH in a national health care system (VA) with a large population of black Veterans, inclusion of chart abstracted data on surgical history and postoperative uterine weight, and an analysis that explicitly recognizes race as a social rather than biologic construct. Nevertheless, our study has several key limitations that need to be considered. First, while our data set is rich in clinical data, it lacks patient-reported outcomes and narratives that would provide critical information on experiences of UF care that could potentially explain our findings. Second, we excluded those with hysterectomies paid for by VA and performed by community providers as pre- and postoperative documentation was not readily available for these surgeries.

Although our earlier work indicates that the likelihood of having a hysterectomy paid for rather than provided by VA does not differ between black and white Veterans, the factors influencing this decision are diverse, and thus it is unclear if our findings are generalizable to those whose hysterectomy is paid for by VA.^19^ Third, this study took place in the context of an integrated national health care system which removes some cost barriers to care, raising concerns regarding generalizability to non-Veterans. However, given that VA providers operate in the same political and social context and receive training in the institutions as non-VA providers, it is likely that many of the same biases are present in non-VA health care.

Finally, our results may not reflect current trends in UF treatment in VA, given rapid ongoing efforts to expand and improve VA gynecology care and growing awareness regarding racial disparities in reproductive health and health care.^28^

Our results have several key implications for policy and practice. Our findings suggest that black/white disparities in MIH are being driven at least, in part, by factors other than fibroid size and number, and this deserves attention and additional investigation. Nevertheless, the higher prevalence of more advanced fibroids among black Veterans compared to white Veterans at the time of hysterectomy, while consistent with findings from other health systems and national data, remains a cause for concern.^3^ To address this fundamental disparity, additional research is necessary to explore the mechanisms linking structural racism to fibroid development, including increased allostatic load, environmental exposures, and modified gene expression,^3,12^ as well as the role of racism in UF treatment and care for Veterans and non-Veterans.

Addressing these root causes requires policies that seek to undo the protracted and harmful impacts of structural racism, including inequities in housing and environmental and occupational exposures that undermine health. Moreover, research that incorporates current experiences of black Veterans with UF care and examines the impact of these experiences on treatment decision-making and outcomes is needed.^48,51^ Such research can be used to develop patient-centered models of care centered on the needs of those who have been historically and systematically mistreated and underserved in the U.S. health care system.^46^ In addition, development of practice guidelines for diagnosis and treatment of UF from national organizations, such as the American College of Obstetrics and Gynecology, that acknowledge and account for the health impacts of racism are needed to improve care for all UF patients.

## Health Equity Implications

Our findings indicate that the association of race and MIH is modified by uterine size as approximated by postoperative uterine weight. These results raise questions about underlying explanations for racial disparities in UF, including the experiences black Veterans have with UF care and treatment in the VA. Specifically, research is needed that seeks to examine upstream causes of UF and racial disparities in MIH that is centered on the perspectives of black patients with UF.

## Abbreviations Used

BMI: body mass index
CDW: Corporate Data Warehouse
CI: confidence interval
FY: fiscal year
ICD-9: *International Classification of Diseases, Ninth Revision*
MIH: minimally invasive hysterectomy
UF: uterine fibroids
VA: Veterans Affairs
